# From Campus to Crisis: Psychological Effect of the COVID-19 Pandemic on Indian Management Students

**DOI:** 10.7759/cureus.57330

**Published:** 2024-03-31

**Authors:** Jallavi Panchamia, Anamika Sinha, Apurvakumar Pandya

**Affiliations:** 1 Health Policy, Management and Behavioural Science, Indian Institute of Public Health Gandhinagar, Gandhinagar, IND; 2 Management, Goa Institute of Management, Goa, IND; 3 Medicine, Parul Institute of Public Health, Parul University, Vadodara, IND

**Keywords:** india, covid-19, psychological well-being, psychological experience, management students

## Abstract

Background: The COVID-19 pandemic has adversely affected students pursuing higher education, but limited studies highlight student’s psychological experiences, especially from Western India.

Objective: The present study aimed to understand psychological experiences, coping behaviors, and the perceived role of tele-counseling services among final-year students of Masters of Business Administration from leading business schools (B-schools) in Western India.

Design: A qualitative research design was adopted for the study. A semi-structured interview guide was utilized to conduct in-depth interviews with 35 students. Students were reached via personal networks and social media, and data was gathered after the second wave of the COVID-19 pandemic. A thematic analysis technique was employed to analyze the data.

Results: The findings showed that management students had upsetting psychological experiences. Key stressors that emerged in the study were job concerns, lifestyle changes, concerns about their own and parents' health and safety, uncertainty about the future, and social isolation. They expressed the need for mental health help; however, they were reluctant to utilize tele-counseling services. The authors present an integrated psychological well-being model for promoting positive mental health among students in higher education institutions.

Conclusion: The study explains the psychological toll on management students. Enhancing mental health literacy through awareness sessions and other innovative means would be critical to addressing myths around mental health and mental healthcare-seeking behavior in higher education institutions. An integrated approach to promoting positive mental health and well-being is needed.

## Introduction

The COVID-19 pandemic has changed educational experiences, mainly how students live, study, and socialize. Public health measures to contain the spread of the coronavirus have forced educational institutes to adopt a new norm of online learning, which has left many flustered. During the lockdown period, in addition to imposed movement restrictions, college students' access to campus and face-to-face classes was suddenly suspended, and internships and research experiences were drastically interrupted [1-3]. Such social isolation due to public health measures reported adverse psychological experiences such as loneliness coupled with accentuated uncertainty toward the future, increased anxiety, anger, fears, depressed feelings, fear of contagion, stigmatization, and many distressing emotions [4-6]. Such adverse psychological experiences impact psychological well-being [7].

Psychological well-being is a combination of positive affective states such as happiness and functioning with optimal effectiveness in individual and social life [8]. Huppert [9] defines psychological well-being as life going well, a combination of feeling good and functioning effectively in day-to-day routine. Psychosocial experience is defined as the phenomenological understanding of experience, which refers to how objects appear in people’s sensing, perception, and meaning-making.

Despite extant literature that has explored the impact of COVID-19 on students’ psychological experiences [10,11], context-specific, demographic-focused studies on college student’s adverse psychological experiences and the state of psychological well-being are limited. Existing studies do not primarily focus on management students' psychological experiences and well-being from India’s premier business schools (B-schools).

Master of Business Administration (MBA) is the most popular degree program B-schools in India offer. Young graduates aspire to earn an MBA to accelerate their careers and bag high-paying jobs [12]. Nearly 300,000 Indians take the entrance exam conducted by the All India Council of Technical Education (AICTE) and the Indian Institute of Management annually, competing for approximately 18,000-20,000 seats in the top 100 colleges [13]. Once admitted to the top B-schools, more than 95% of job placement is almost guaranteed, with an average salary of more than INR 10 lakhs per annum [13].

The COVID-19 impacted the corporate sector, leading to uncertainty in B-school graduates' job prospects. More than one million MBA students graduated in April, with the lockdown period starting from March 2020, unsure of the business response and sectoral impact of the lockdown on business revival and continuity. Premium B-schools charge hefty fees and promise a dramatic improvement in employability, thus adding complexity. It is well known that if someone compromises their initial joining salary due to recession, their career will suffer for a long time, even after revival [14]. Students have faced a substantial psychological toll, and a support system is inevitable to build resilience and help students cope with adverse psychological experiences. Research revealed that B-school students undergo stress due to academic and non-academic demands during the regular course of the two-year program [15]. In addition, students experience stress due to homesickness, interpersonal issues with classmates, financial burdens, and anxiety related to future jobs [16].

Evidence indicates that approach coping mechanisms lead to a cognitive, emotional, or behavioral shift to engage actively with stressful situations. Although there is a limited reach of technology in the mental health domain [17], technology-enabled interventions have gained immense importance during and after the COVID-19 pandemic. According to Roth, long-term positive outcomes can be facilitated through counseling and support systems [18]. Few studies have been conducted to understand the usage of online technological interventions in improving mental health, but they do not address the context of management students in India and specifically the perceived role of tele-counseling [19,20]. In this context, the objective of the present study was to explore the psychological experiences and coping behaviors of management students and the perceived role of tele-counseling among management students in India.

## Materials and methods

This study employed a qualitative approach using semi-structured interviews for data collection. Data were collected from three schools in Western India [21]. Students were selected from the Goa Institute of Management, the Indian Institute of Public Health, and Parul University. The study sample included students from the final year of postgraduate management studies. Students who were pursuing their postgraduate management studies as part-time or evening MBA and had a non-engineering background were excluded from the study sample.

A semi-structured interview protocol was developed and pilot-tested. The majority of the key questions were derived from a finding of the previous study by Renk and Smith [22] on college students. The data was collected in the period from October to December 2021. Students were contacted through social media and personal networks. Using purposive and snowball sampling techniques, 35 students were telephonically interviewed. The average time for the interview was 20 minutes. The 30th interview reached data saturation, but we interviewed more people to get deeper insights.

The questions asked are given in Table 1. A total of 11 questions were asked on various aspects including their experiences, concerns, social media usage, and perception toward counseling.

**Table 1:** The structure of the interview questions Source: These questions were developed by the authors.

S. No.	Interview Questions
1	Briefly describe your experience during the lockdown period.
2	What were the top stressors before lockdown as an MBA student?
3	What are your top concerns during the lockdown and COVID pandemic?
4	What activities and behaviors are you using to keep yourself occupied and being able to cope with the current situation?
5	How much time were you spending on social media pre-COVID?
6	How much time were you spending on social media post-COVID?
7	How much time in a week are you devoting to exercise, health, and fitness?
8	How would you describe your stress experience during the lockdown?
9	What is your perception toward counseling?
10	Have you considered a need for counseling/coaching during this period?
11	Were you seeing the counselor before the lockdown?

Construct validity was ensured by collecting multiple data points. The questionnaire hierarchy was generated to ask interlinked and related questions. A chain of evidence was established through data collection, analysis, interpretation, and checking for existing evidence in the literature available. Further, we looked for patterns (if any), and they were analyzed and matched to previously reported patterns under similar contexts. The results indicated coping strategies of avoidance and approach in the dynamic interactions of parenting, social support system, academic demands, and other emotional and problem-centered demands.

All participants were informed about voluntary participation and gave their informed consent before participating in the study. Students were informed of the purpose of the research inquiry. They were also assured of confidentiality and mental health support availability if a need emerged during as well as after the conversation.

The data was analyzed using thematic analysis techniques [23]. The researchers read the transcripts and registered the repeating word counts. These words were then put in buckets of similar themes. Detailed sample narratives on the emerging themes were then chosen to build an understanding of the phenomenon. The first two researchers did this process individually and then triangulated the narratives. Further, researchers looked for themes. Emerging themes were analyzed and matched to previously reported themes from the literature under similar contexts.

## Results

Participants were in their final year of postgraduate management studies with an average age of 25 years. These students had pursued engineering. After earning their engineering degree, they had up to five years of work experience in Information Technology-Enabled Services (ITES), Fast Moving Consumer Goods (FMGC), and consulting sectors. In the sample, five students had no prior work experience. Gender distribution was uneven, with 10 female students and 25 male students (Table 2). From the data presented below, the themes emerged, concerns regarding job concerns, financial concerns, health and safety concerns, lifestyle concerns, psychological distress, constructive anchors, and coping actions (Table 3).

**Table 2:** Demographic and educational status of respondents (N = 35)

Demographic variables	N	%
Gender		
Male	25	71.4%
Female	10	28.6%
Age		
22 to 24 years	22	62.9%
25 to 27 years	08	22.9%
28 to 30 years	05	14.3%
Field of management studies		
Hospital management	12	34.3%
Business management	14	40.0%
Public health management	9	25.7%
Years of experience		
1-3 years	19	54.3%
4-5 years	11	31.4%
No previous experience	05	14.3%

**Table 3:** Thematic analysis of key results

Codes	Frequency	Sub-theme	Theme
Security, retrenchment, loss	35	Job insecurity, financial well-being	Job concerns
Delayed/unclear joining date	20		
Parents wellness	15	Concern regarding the safety and health of self and loved ones	Health and safety concerns
Self-health	20		
Loved ones	25		
Miss campus life and friends	15	Concerns emerging from changed lifestyle	Lifestyle concerns
Uncertainty	16		
Feeling anxious	12		Psychological distress
Boredom	12		
Feeling depressed	5		
Loneliness	7		
Stuck	5		
Anger, clutter, confusion	>5		
Time with family	20	Positive and constructive anchors among students	Constructive anchors
Hope, gratitude, kindness, relaxation	>5		
Upskilling	20	Action and coping behavior of students with stress	Coping strategies
Media and screen time	8		
Networking	6		
Mental health support	>5		

Job concerns

The students were concerned about whether they could bag their dream jobs and have financial security. Even if they do, would they be remunerated following the "generic" norms and standards, given that the economy has gone into recession? One of the participants (a 27-year-old male student) stated, *"Newspaper and media are abundant with news on job cuts, delayed onboarding and joining, companies withdrawing their offers, etc. The pandemic coincides with the academic calendar of March to March. Corporates have extended joining for both summers and final placement. The future seems uncertain. One will know the exact situation later".*

Another participant (a 24-year-old female student) said, "*Certain recruiters from industries like infrastructure, hospitality, travel, retail, logistics, automobiles, and entertainment will take time to revive, impacting salary and jobs. There is a possibility of salary adjustments, digitalization of work, Gig workforce, flexible work, and work from home. These eventualities and lack of adequate preparedness are overwhelming*."

Financial concerns

Yet another narrative from a participant indicated their financial concern for repayment of loans. Most students secured educational loans. B-school has been synonymous with a guaranteed job, but with the pandemic, students are concerned about getting a job. A participant (a 29-year-old male student) expressed, "*…an MBA in India from a leading B-school could cost anywhere between 20,00,000 and 25,00,000 Lakhs. Most students are on partial or full loans ranging from 10 to 15 years at an interest rate of 9%. The minimum equal monthly installment of loan repayment is INR 17,000 per month. If timely payment is missed, the bank will add a moratorium. This adds to the stress. [Moreover] our parents have retired or nearing retirement. Some parents are dependent on savings... [Therefore] in the COVID-19, we were worried about the job that can enable us to repay the loan and provide financial support to the family..."*

Health and safety concerns

The following concern by the participants was the health and safety of self, family, and loved ones. A male participant (25 years old) shared, "*I got anxious when I saw the news related to people affected by the virus and related mortality. I did not want to see my parents and family suffer."*

Some participants shared similar thoughts that it would be okay if they fell sick, but it would be tough to manage any of their parent's sicknesses. A verbatim by one of the participants (a 27-year-old female) reflects this, "*I get tensed when I see the elderly getting infected and even dying. This situation creates a lot of anxiety.*"

Importantly, participants started taking care of their own and parents' health as they realized the importance of being healthy. One participant (a 25-year-old female) said, "*I ensure that I am working toward a healthy lifestyle which I was so far taking for granted."*

Lifestyle concerns

Students underwent lifestyle changes due to the lockdown period since they were compelled to stay home. They had to take online classes and were away from their campus life, fellow batch mates, faculties, and in-person group work. This change led to various emotions and frustration. A participant (a 23-year-old male student) said, "… *during the lockdown, staying back at home was difficult. Most of my friends were not following the strict quarantine norms and were interacting. I did not come out as my parents are co-morbid. Staying at home has been lonely and boring."*

Another respondent (a 27-year-old male student) mentioned, "*I have been away from home since graduation, so readjusting with my parents is difficult. They have stopped looking at me since I left, but I have grown up. On the contrary, I see them getting older and weaker. This leads to conflicts and often misunderstandings. Our roles have changed. I am no longer the baby that needed care.*"

Uncertainty of the future and social isolation

Participants experienced psychological distress in terms of the experience of anxiousness, depressed feelings, anger, and frustration due to the uncertainty of the future. The pandemic-imposed lockdown resulted in a loss of activity, interactions, and loss of campus buzz. These become a source of loneliness, boredom, and depression. A participant (a 24-year-old female student) expressed, "*I missed campus and campus life during lockdown. I was uncertain about life and felt disconnected. All co-curricular activities, group learning, and group work were significantly compromised, making us feel inadequately ready for the future. It was difficult to adjust to this new normal life.*"

Constructive anchors

Some participants felt grateful for being able to retain their jobs, being with family, and being in good health, while many others struggled. As one of the participants (a 26-year-old male student) expressed, "*Thank God I was here with my family and able to help them during such a difficult period. It might have created more stress for me if I had been in a hostel.*"

A participant (a 24-year-old female student) also shared, "*I always used to crave some good time with my mom, but this lockdown period gave me that opportunity to help her with household chores and other fun stuff in the kitchen. I came much closer to my father too, as I spent hours in various kinds of discussion with him daily."*

Coping strategies

Students expressed watching Netflix or other over-the-top (OTT) programs to use the time available. Time spent on social media and television accounted for four to 10 hours daily. A participant (a 23-year-old male student) mentioned, "*My parents had never watched Netflix. But now even my mother watches Netflix with me. In some ways, that has emerged as the family bonding time."*

Some mentioned that they were upskilling and pursuing various virtual learning programs. The average time spent on this was about two hours per week. Virtual courses were important for many because they wanted to utilize their free time positively. At the same time, some focused on improving immunity by engaging in physical activities (exercising) at least for 30 minutes to one hour daily, which is echoed in the following verbatim. "*We have to exercise, as we were home the whole day and were not going out during lockdown. Our junk food has increased. Chips and biscuits are all adding, and I have also gained weight and become lethargic." *said a 23-year-old male student.

Most participants agreed to face psychological distress and perceived the need for mental health support; however, (surprisingly!) they were reluctant to take help through tele-counseling platforms. A participant (a 22-year-old female student) said, *"Sometimes we feel like talking to someone who would understand. There is a need for a support system for mental health. But for whatever reasons, I have not called the counselors.*"

The following excerpts reflect students' non-acceptance of tele-counseling. A 25-year-old male student said, "*I had been seeing the therapist earlier than the lockdown, so while tele-counseling was not something I looked forward to, I approached the counselor online."*

Those who were appraised with the concept of counseling were more open to tele-counseling and seeking mental health help. For example, one participant (a 27-year-old female student) said, "…*the comfort with tele-counseling was lesser, but given that that was the only option available during the lockdown, I did reach out to the counselor and sought help."*

These narratives indicate differing levels of acceptance toward tele-counseling. Different experiences were available from fear to acceptance to adjusting to a new approach.

## Discussion

The findings indicate the changing nature of stressors. The students were questioning many things that a management degree otherwise implicitly promised. New dimensions of stress were added, like finding a job and repaying loans, health safety for self and care for the family members, changed lifestyles, and confusion (especially regarding what to prioritize in their life at the moment). Further, they also experimented with positive anchors and coping methods during difficult times.

Worrying about getting a job and financial well-being has been reported in the literature [24-26]. Mujtaba et al. [27] noted that mental stress due to fear of job loss leaves a long-lasting mark on the individual's mental health. Under recessionary threats, employees negotiate salaries lower than the prevailing market rates, which takes a long time to get back at par regarding expected career growth. Notably, the effect of personal debt and fear of one's financial well-being are known to affect mental health to a great extent.

Health and safety of self and loved ones were also reported in the study. COVID-19 has changed the student's perspective to emerge as a care provider rather than just a care seeker [27]. Lifestyle change was a significant experience shared by participants in the present study. Previous studies have also reported similar findings. For example, stress from adjustment to campus life, including interpersonal relations and peer pressures, is well recorded [28]. Participants unanimously expressed quality time and communication with family as a source of family resilience.

**Figure 1:**
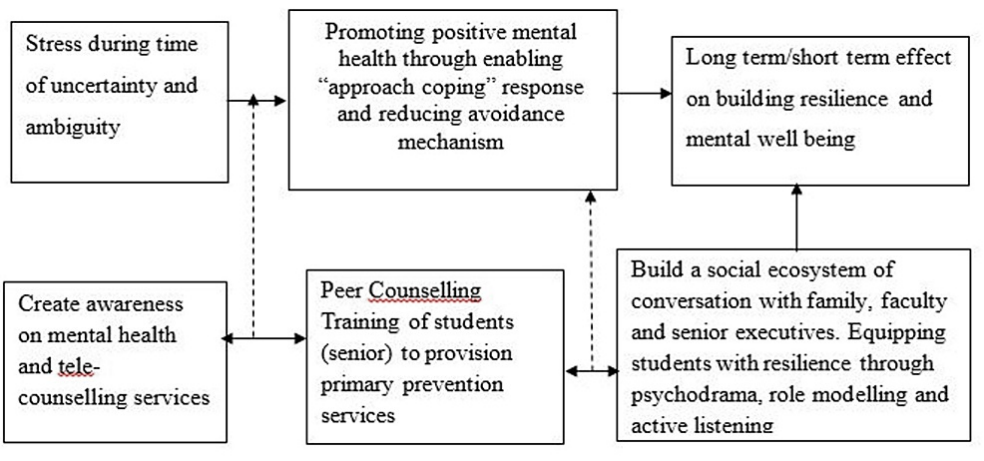
Proposed integrated psychological well-being model Image credits: Authors of this study.

To deal with emergent demands, we propose an integrated psychological well-being model to promote positive mental health. Figure 1 depicts a schematic presentation of the model. Findings revealed coping responses ranging from consuming junk food, over-usage of social media/OTT streaming, over-sleeping, watching television, spending time with family, exercising, and upskilling to reduce stress. Some of these coping responses were avoidant, while some were approach coping behaviors [29]. Students need to be guided to utilize approach coping as a strategy to overcome psychological toll. To extend psychological support to college-going students, the Government has introduced tele-counseling services. However, many students still need to choose it as a support mechanism. Therefore, busting myths related to tele-counseling through mental health awareness sessions, strategic behavior change communication to destigmatize mental health, and promoting mental health ambassadors (positive role models) are needed more prudently during health emergencies like COVID-19.

Students need an explanation of the industry's changing contexts and dynamics, and helping with information on emerging areas of development and upskilling could help students immensely [30]. The B-school students were unprepared for such an academic environment and probable coping mechanisms. Through dialogues and behavioral shifts, the students could be motivated to reach out and remain in touch with a counselor who could help students remain hopeful, optimistic, and focused on their careers. It is also important for B-school authorities as the B-schools were also unprepared for such an academic environment and probable coping mechanisms.

Training college students in peer counseling is a viable strategy. D'Andrea discussed the peer counseling concept in 1987 [30]. It is defined as a helping process that involves a one-on-one conversation between a homogenous group. In an academic setting, it refers to trained students helping their peers. It is a way of relating, responding, and helping people, exploring thoughts, feelings, issues, and concerns, hoping to reach a clear understanding and making informed decisions. As a result of peer counseling support, individuals may develop skills in (1) identifying and labeling stress/anxiety and understanding the response to the trigger that is causing the stress (avoidance or approach), (2) developing intrusive alternate thoughts, (3) engaging in conversations about the trauma and sharing stories about what is happening to them, and (4) converting the story or experience as an event like a fork at the end of a road where grief and gratitude, strengths and vulnerabilities, and a reminder on how those strengths were used as a resource by the individual in the past can be developed [30].

Peer counseling also enables the provisioning of psychological first-aid by trained fellow students. Psychological first-aid is a "humane, supportive response to a fellow human being suffering and may need support" [31]. It is intended as short-term emergency care by listening and rephrasing the stressful event as an opportunity for caregivers. It helps an individual to feel safe, connected, and supported and builds a positive emotional contagion. In this context, peer counseling training can include psychological first-aid to create a responsive ecosystem in the academic setup.

As revealed in the study, several students were experiencing distressful psychological experiences. Despite needing professional help, many still need to access tele-counseling services. One possible reason might be a lack of confidence in the effectiveness of tele-counseling over one-on-one interaction, fear that counselors may need help understanding their problems, and negative experience of seeking tele-counseling service. This highlights the need to create mental health literacy (especially addressing myths related to tele-counseling) and upskill tele-counselors' professional skills. Tele-counseling will need interventions at three levels to promote health-seeking behaviors among students. (a) Train tele-counselors on virtual empathy and effective ways of communicating with students; (b) actively communicate about the importance of professional help in fostering positive mental health through several mediums, and (c) sensitize them with available sources of help. While the efficiency of these tele-counselors, even with training, needs to be established, contradicting perspectives are available. Hyun et al. [32] reported lower openness to tele-counseling in stressful situations. Therefore, creative ways of engaging with the students through virtual empathy must be developed.

## Conclusions

The study revealed management students' perceived need for professional help and highlighted their disturbing psychological experiences. Key stressors were job concerns, lifestyle change, concerns about their and their parent's health and safety, uncertainty about the future, and social isolation. They also expressed the need for mental health help but were reluctant to utilize tele-counseling services.

The proposed integrated psychological well-being model will be an alternative support system in an emerging nation like India, where mental health professionals are limited. The need for a “task shifting” approach is evident, and the integrated model attempts to bridge this gap. The inherent limitation of qualitative research is its generalizability. While this study explains the phenomenon of psychological experiences and coping behavior in the sample, the findings remain contextual with limited generalizability. Future research should study the theoretically proposed model with a large sample.

## References

[1] Aras B, Insung J, Junhong X (2020). A global outlook to the interruption of education due to COVID-19 pandemic: navigating in a time of uncertainty and crisis. Asian Journal of Distance Education.

[2] Pandya A, Lodha P (2021). Mental health of college students amidst COVID-19: implications for reopening of colleges and universities. Indian J Psychol Med.

[3] Pandya A, Lodha P (2022). Mental health consequences of COVID-19 pandemic among college students and coping approaches adapted by higher education institutions: a scoping review. SSM Ment Health.

[4] Cao W, Fang Z, Hou G, Han M, Xu X, Dong J, Zheng J (2020). The psychological impact of the COVID-19 epidemic on college students in China. Psychiatry Res.

[5] Roy D, Tripathy S, Kar SK, Sharma N, Verma SK, Kaushal V (2020). Study of knowledge, attitude, anxiety and perceived mental healthcare need in Indian population during COVID-19 pandemic. Asian J Psychiatr.

[6] Son C, Hegde S, Smith A, Wang X, Sasangohar F (2020). Effects of COVID-19 on college students' mental health in the United States: interview survey study. J Med Internet Res.

[7] Tan BY, Chew NW, Lee GK (2020). Psychological impact of the COVID-19 pandemic on health care workers in Singapore. Ann Intern Med.

[8] Ryff CD (2014). Psychological well-being revisited: advances in the science and practice of eudaimonia. Psychother Psychosom.

[9] Huppert FA (2009). Psychological well‐being: evidence regarding its causes and consequences. Applied Psychology: Health and Well-Being.

[10] Ma H, Miller C (2021). Trapped in a double bind: Chinese overseas student anxiety during the COVID-19 pandemic. Health Commun.

[11] Elmer T, Mepham K, Stadtfeld C (2020). Students under lockdown: comparisons of students' social networks and mental health before and during the COVID-19 crisis in Switzerland. PLoS One.

[12] Ray A, Bala PK, Dasgupta SA, Srivastava A (2020). Understanding the factors influencing career choices in India: from the students' perspectives. Int J Indian Cult BU.

[13] Verma Verma, P. & Bhattacharya, S. (2020, Feb 24 (2020). It pays to do an MBA in a slowdown. https://economictimes.indiatimes.com/jobs/placements-for-the-class-of-2020-non-bluechip-b-schools-beat-slowdown-blues/articleshow/74234601.cms.

[14] Oreopoulos P, Von Wachter, Heisz A (2012). The short-and long-term career effects of graduating in a recession. American Economic Journal: Applied Economics.

[15] Fairbrother K, Warn J (2003). Workplace dimensions, stress and job satisfaction. J Manag Psychol.

[16] Sinha A (2014). Stress vs academic performance. SCMS Journal of Indian Management.

[17] Witt KJ, Oliver M, McNichols C (2016). Counselling via avatar: professional practice in virtual worlds. Int J Adv Couns.

[18] Roth T (2022). The Stress-Buffering Effect of Receiving Social Support on Resilience in Families of Children With Medical Complexity. https://www.proquest.com/openview/fcd1ca836a6ece2a503340e63ff6f5bd/1?pq-origsite=gscholar&cbl=18750&diss=y.

[19] Kazdin Kazdin, Alan E (2015). Technology-based interventions and reducing the burdens of mental illness: perspectives and comments on the special series. Cogn Behav Pract.

[20] Figueroa CA, Aguilera A (2020). The need for a mental health technology revolution in the COVID-19 pandemic. Front Psychiatry.

[21] Pordelan Pordelan, N. N., & Hosseinian, S. S. (2021). They mean business. https://www.indiatoday.in/magazine/india-s-best-b-schools/story/20181126-they-mean-business-1389742-2018-11-17.

[22] Renk K, Smith T (2007). Predictors of academic-related stress in college students: an examination of coping, social support, parenting, and anxiety. Naspa Journal.

[23] Braun V, Clarke V (2006). Using thematic analysis in psychology. Qualitative Research in Psychology.

[24] Bhuiyan AK, Sakib N, Pakpour AH, Griffiths MD, Mamun MA (2021). COVID-19-related suicides in Bangladesh due to lockdown and economic factors: case study evidence from media reports. Int J Ment Health Addict.

[25] Frasquilho D, Matos MG, Salonna F, Guerreiro D, Storti CC, Gaspar T, Caldas-de-Almeida JM (2016). Mental health outcomes in times of economic recession: a systematic literature review. BMC Public Health.

[26] Fitch C, Hamilton S, Bassett P, Davey R (2011). The relationship between personal debt and mental health: a systematic review. Ment Health Rev J.

[27] Mujtaba BG, Knapp P, Baker D, Ahmed MR (2009). Stress overload perceptions of American MBA students in recessionary times. The IUP Journal of Organizational Behavior.

[28] Sinha A, Panchamia J, Sachan A, Guru S. (2021). Choice of electives among management students in India: A conjoint analysis. The. Int J Manag Educ.

[29] Reddy KJ, Menon KR, Thattil A (2018). Academic stress and its sources among university students. Biomed Pharmacol J.

[30] D'Andrea VJ (1987). Peer counselling in colleges and universities: A developmental viewpoint. J Coll Stud Psych.

[31] Khawaja NG, Stallman HM (2011). Understanding the coping strategies of international students: a qualitative approach. Aust J Guid Couns.

[32] Hyun J, Quinn B, Madon T, Lustig S (2007). Mental health need, awareness, and use of counseling services among international graduate students. J Am Coll Health.

